# Aetiology of acute meningoencephalitis in Cambodian children, 2010–2013

**DOI:** 10.1038/emi.2017.15

**Published:** 2017-05-24

**Authors:** Paul F Horwood, Veasna Duong, Denis Laurent, Channa Mey, Heng Sothy, Ky Santy, Beat Richner, Seiha Heng, Sopheak Hem, Justine Cheval, Christopher Gorman, Philippe Dussart, Menno D de Jong, Alexandra Kerleguer, Bertrand Guillard, Bernadette Murgue, Marc Lecuit, Xavier de Lamballerie, Jeremy J Farrar, Arnaud Tarantola, Marc Eloit, Philippe Buchy

**Affiliations:** 1 Virology Unit, Institut Pasteur in Cambodia, Phnom Penh 12000, Cambodia; 2 Kantha Bopha Hospital, Phnom Penh 12000, Cambodia; 3 Medical Laboratory, Institut Pasteur in Cambodia, Phnom Penh 12000, Cambodia; 4 PathoQuest SAS, Paris 75013, France; 5 Department of Medical Microbiology, Academic Medical Center, University of Amsterdam, Amsterdam 1012, The Netherlands; 6 World Health Organization, Geneva 1211, Switzerland; 7 Biology of Infection Unit, Institut Pasteur, Inserm U1117, Paris 75015, France; 8 Aix Marseille Université, IRD French Institute of Research for Development, EHESP French School of Public Health, Marseille 35043, France; 9 Oxford University Clinical Research Unit, Wellcome Trust Major Overseas Programme, Ho Chi Minh City P1Q5, Vietnam; 10 Epidemiology & Public Health Unit, Institut Pasteur in Cambodia, Phnom Penh 12000, Cambodia; 11 GlaxoSmithKline Vaccines Asia-Pacific, Singapore 189720, Singapore

**Keywords:** chikungunya virus, dengue virus, encephalitis, Japanese encephalitis virus, meningoencephalitis, meningitis, paediatric, scrub typhus

## Abstract

Acute meningoencephalitis (AME) is associated with considerable morbidity and mortality in children in developing countries. Clinical specimens were collected from children presenting with AME at two Cambodian paediatric hospitals to determine the major aetiologies associated with AME in the country. Cerebrospinal fluid (CSF) and blood samples were screened by molecular and cell culture methods for a range of pathogens previously associated with AME in the region. CSF and serum (acute and convalescent) were screened for antibodies to arboviruses such as Japanese encephalitis virus (JEV), dengue virus (DENV), and chikungunya virus (CHIKV). From July 2010 through December 2013, 1160 children (one month to 15 years of age) presenting with AME to two major paediatric hospitals were enroled into the study. Pathogens associated with AME were identified using molecular diagnostics, cell culture and serology. According to a diagnostic algorithm, a confirmed or highly probable aetiologic agent was detected in 35.0% (*n*=406) of AME cases, with a further 9.2% (total: 44.2%, *n*=513) aetiologies defined as suspected. JEV (24.4%, *n*=283) was the most commonly identified pathogen followed by *Orientia tsutsugamushi* (4.7%, *n*=55), DENV (4.6%, *n*=53), enteroviruses (3.5%, *n*=41), CHIKV (2.0%, *n*=23) and *Streptococcus pneumoniae* (1.6%, *n*=19). The majority of aetiologies identified for paediatric AME in Cambodia were vaccine preventable and/or treatable with appropriate antimicrobials.

## INTRODUCTION

Meningitis and encephalitis are infections of the meninges and brain, respectively, and are major causes of mortality and long-term neurological sequelae, particularly in children throughout the developing world. As these illnesses are difficult to clinically diagnose, they are often grouped under the term acute meningoencephalitis (AME). AME is among the most frequent and severe causes of pediatric hospitalization across Asia.^1^

Bacterial meningitis is a serious condition; about 10% of patients die even with adequate and timely antibiotic treatment, whereas the fatality rate can rise to 40%–58% when access to prompt treatment is unavailable.^2^ Many children who survive the infection are left with permanent neurological impairments such as hearing loss, learning disabilities, and behavioural problems.^3^ The burden of these sequelae is unknown. It has been estimated that 16 million cases of acute bacterial meningitis, resulting in approximately 300 000 deaths, occur throughout the world each year, the vast majority in developing countries.^4, 5^ *Haemophilus influenzae* type b, *Streptococcus pneumoniae* and *Neisseria meningitidis* account for ~80% of reported cases of bacterial meningitis.^4^

The viral agents of meningitis and encephalitis vary greatly between countries, with herpesviruses being the major aetiology in developed countries and vector-borne pathogens being dominant in tropical developing countries.^6^ Japanese encephalitis virus (JEV) has been reported as the main cause of AME in Southeast Asia, with a conservative estimate of 68 000 cases occurring annually.^7, 8^ Children in developing countries bear the greatest burden of morbidity and mortality from AME infections, particularly in Southeast Asia where important encephalitic pathogens such as JEV, enterovirus 71 (EV-A71) and dengue virus (DENV) commonly circulate.

Cambodia (population 15.1 million) is one of the poorest countries in Asia (Gross Domestic Product per capita of $1100 USD per annum).^9^ There has been no systematic report published about the causes of AME in Cambodian children, although it is thought that JEV, DENV and tuberculosis are common aetiologies.^10, 11^ Due to the absence of sufficient diagnostic capacity in most hospitals, Cambodian clinicians commonly treat AME empirically with little evaluation of the effectiveness of their clinical approach. The aim of this study was to better define the major aetiologies of paediatric AME in Cambodia through a prospective hospital-based study from 2010 to 2013.

## MATERIALS AND METHODS

### Patients and clinical specimens

Kantha Bopha hospitals represent the largest paediatric hospital network in Cambodia, with an estimated 90% of all hospital care delivered to Cambodian children in these hospitals. The two major paediatric hospitals in the Southwest and Northeast of the country were included in the study: Kantha Bopha Children’s Hospital in Phnom Penh and Jayavarman VII Hospital in Siem Reap, respectively.

Children were admitted into the study from July 2010 until the end of December 2013. Each week, an average of five children were randomly selected to be enroled into the study among patients aged >1 month and <15 years who presented with clinical status that required a lumbar puncture for which the admission diagnosis was AME. Basic demographic information was collected from all patients using case report forms. AME was defined as the presence of the following criteria:

- Fever >38 °C, or febrile episode reported within the previous month.
- Cerebrospinal fluid (CSF) abnormalities (>four white blood cells per mm^3^ or CSF proteins >0.4 g/L).
- At least one of the following signs: confusion; prolonged, altered consciousness; seizures; central neurological deficiency.

The study started with Jayavarman VII Hospital in Siem Reap in July 2010, and in order to have a better representativeness of the epidemiology of AME in the country Kantha Bopha Hospital in Phnom Penh was included during 2013. The hospital in Phnom Penh was not included in early recruitment of cases as the hospital was not equipped with a magnetic resonance imaging system until 2012.

For virological and microbiological testing, the following specimens were obtained during the first two days of admission: EDTA-anticoagulated blood (2 mL minimum); clotted whole blood (1 mL); CSF (2 mL collected during routine investigations); throat and rectal swabs in viral transport medium (VTM). Blood and CSF culture for bacteria isolation was also performed onsite in the hospitals according to standard procedures. All samples were collected from patients based on the medical judgement of individual clinicians as part of the patient’s care management process. Specimens were kept at 4 °C after sampling and transported daily to the laboratory at the Institut Pasteur du Cambodge. Upon arrival in the laboratory, the specimens were immediately processed and frozen at −80 °C until further analysis.

### Molecular biology testing

Nucleic acids were extracted from all clinical samples using the MagNa Pure LC system (Roche Life Science, North Ryde, NSW, Australia), according to the manufacturer’s instructions. Previously described PCR/RT-PCR (real-time and conventional) methods were used to screen for a large range of viral and bacterial pathogens (Supplementary Table S1), including the viruses: JEV, DENV1-4, chikungunya virus (CHIKV), flavivirus universal, EV-A71, enterovirus universal, influenza A viruses, Epstein-Barr virus (EBV), cytomegalovirus (CMV), herpes simplex virus (HSV), varicella-zoster virus (VZV); and the bacteria: *S. pneumoniae*, *H. influenzae*, *N. meningitidis*, *Orientia tsutsugamushi* and *Streptococcus suis*. *Mycobacterium tuberculosis* testing was also undertaken using GeneXpert for the first 40 patients enroled with a high clinical suspicion of tuberculosis AME, but testing was discontinued due to the requirement for high volumes of CSF and the absence of positive results. Testing of other pathogens (for example, rabies, measles and mumps) was conducted if the clinical presentation was suggestive of a particular infection.

### Serological testing

All acute sera, convalescent sera and CSF samples were tested for anti-JEV, -DENV and -CHIKV IgM using in-house capture enzyme-linked immunosorbent assays (MAC-ELISA) as originally described by Rossi and Ksiazek^12^ and adapted for our laboratory as outlined previously.^13, 14^ A result was considered positive when the optical density (OD) of the sample was greater than the mean OD of three negative control samples plus three standard deviations. An acute infection was defined as an IgM seroconversion or a significant increase of the OD measured between admission and discharge sera. To rule out the possibility of contamination of the CSF with blood IgM during sampling, CSF samples were only considered positive when the OD was higher than the respective blood IgM OD reading. In cases where both JEV and DENV IgM were detected positive the result was recorded as positive for undifferentiated flavivirus.

### Cell culture isolation

All CSF and serum samples that tested positive by PCR or serology for JEV, CHIKV, DENV and flaviviruses were inoculated onto C6/36 (*Aedes albopictus*) cells in order to isolate the virus for subsequent analysis. In addition, CSF was inoculated onto Vero E6 (African green monkey kidney epithelial) cells from all patients when there was sufficient CSF samples remaining after molecular and serological testing.

### Testing algorithm and decision tree

**Figure 1:**
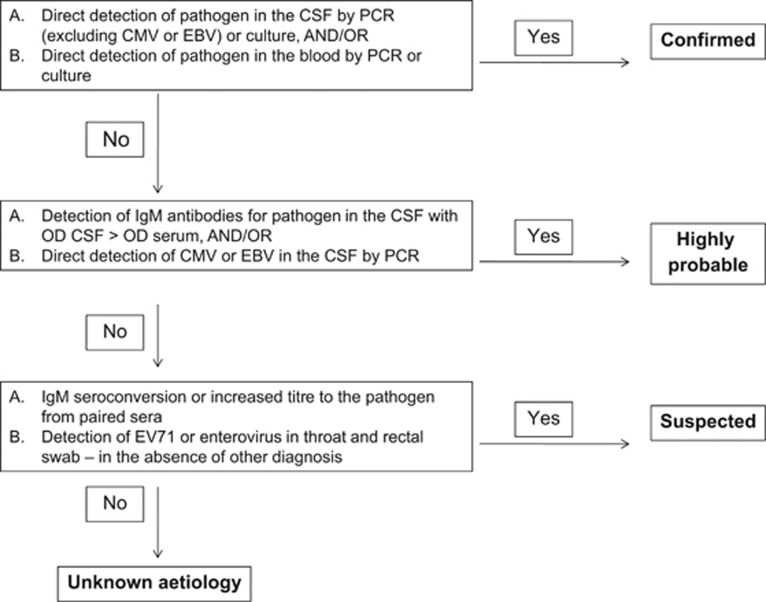
The diagnostic algorithm used for the study.

**Figure 2:**
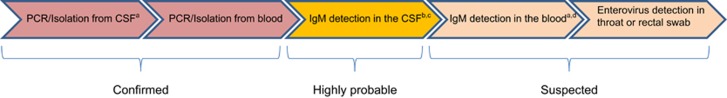
A decision tree used in the study to prioritize the importance of results in determining the final conclusion for aetiology. Ranking of the primacy of results is from left (most reliable) to right (least reliable). ^a^Detection of CMV or EBV in the CSF was categorized as ‘highly probable’ due to the possibility of reactivation of latent virus. ^b^IgM detection was considered positive when the optical density (OD) of the samples was greater than the mean OD of three negative control samples plus 3 standard deviations. ^c^IgM detection in the CSF was only considered positive when the CSF IgM titre was higher than blood IgM titre for the corresponding virus. ^d^An acute infection was defined as a significant increase in OD measured for IgM by ELISA between admission and discharge sera.

A diagnostic algorithm (Figure 1) was designed to define the aetiological link between the detection of pathogens using different methodologies. Aetiology was considered ‘confirmed’, ‘highly probable’ or ‘suspected’ according to the criteria outlined in Figure 1. Aetiology was classified as unknown for cases in which all tests were negative. In cases where there was conflicting results between different tests a decision tree (Figure 2) was used to decide on the final aetiology of the AME.

We defined the molecular detection of CMV and EBV in the CSF as a ‘highly probable’ aetiology due to the unknown frequency in which these viruses can reactivate from latent infection and be detected in the CSF without being the cause of AME. However, only cases where virus was detected above a threshold of 100 copies were included as positive aetiologies.

### Representation and mapping of JEV cases

Clinical data were entered using Excel (Microsoft Corp, Redwoods, WA, USA) and explored by province of patient residence using STATA 11 (StataCorp, College Station, TX, USA). Confirmed cases were represented by month in a graph using Excel along with mean rainfall data estimated by satellite teledetection (Tropical Rainfall Measuring Mission, NASA, USA, http://trmm.gsfc.nasa.gov/) and adjusted for surface of province of residence. Population-adjusted attack rates (cases per 100 000 pop, using 2012 population) for each study period (2010–2012 and 2013) were mapped using ArcGIS 10 (Esri, Redlands, CA, USA), by year and patient’s province of residence, to assess the time and geographical distribution of all ‘confirmed’ and ‘highly probable’ JEV cases for which sufficient information for date of hospitalization and location of residence were recorded.

### Next-generation sequence analysis

Following revision of the case report forms, patients for whom an aetiology was not identified, but clinical evidence was suggestive of a viral infection, were selected for next-generation sequence (NGS) analysis. CSF samples, sera and nasopharyngeal swabs from representative patients (*n*=13) were sent to PathoQuest SAS (Paris, France) for NGS analysis using an Illumina HiSeq-2000 sequencer (DNAVision, Gosselies, Belgium). Sample preparation, sequencing and bioinformatic analysis was conducted as described in Gagnieur *et al*^15^ except that human genome sequence subtraction was done by reference to NCBI build 37.1/assembly hg19.

### Ethical approval

This study was approved by the Cambodian National Ethics Committee for Human Research (approval #107NECHR and #212NECHR). All samples were collected after obtaining informed consent from the patient’s parents or guardians. Informed consent was also obtained for all patients to send samples internationally for NGS analysis.

## RESULTS

A total of 1160 paediatric AME cases were enroled: 930 (80.1%) patients from Jayavarman VII Hospital in Siem Reap (from July 2010 through September 2013) and 229 (19.9%) patients from Kantha Bopha Hospital in Phnom Penh (from February 2013 through December 2013). The demographic characteristics of the patients included in the study are outlined in Table 1 and a summary of the biochemical tests used to inform the diagnosis of AME is provided in Supplementary Table S2.

An infectious aetiology was detected in 35.0% (*n*=406) of AME cases enroled in the study using the criteria for assigning ‘confirmed’ and ‘highly probable’ cases (Table 2). When ‘suspected’ cases were also included, the proportion of cases with an aetiology identified was 44.2% (*n*=513). JEV was the most commonly detected pathogen with 20.3% of cases categorized as ‘confirmed’ or ‘highly probable’ and a further 4.1% defined as ‘suspected’ (total 24.4%, *n*=283). Other pathogens frequently associated with AME included *O. tsutsugamushi* (4.7%, *n*=55), DENV1-4 (4.6%, *n*=53), enterovirus/EV-A71 (3.5%, *n*=41), CHIKV (2.0%, *n*=23) and *S. pneumomiae* (1.6%, *n*=19). Conflicting results were detected in 16 patients and included instances where two or more pathogens with a classification of ‘confirmed’ or ‘highly probable’ were detected. Patients with conflicting results also included five cases where two or three pathogens were detected in a patient’s CSF by molecular testing. These results were included in the aetiology counts (Table 2), but these cases were also included in Supplementary Table S3 for further scrutiny.

NGS analysis of the CSF with negative targeted screening did not result in the detection of any pathogens in AME patients. NGS analysis of nasopharyngeal swabs from representative patients (*n*=13) resulted in the detection of common respiratory viruses in some samples (human parainfluenza virus 4, human metapneumovirus, human respiratory syncytial virus). Targeted real-time RT-PCR testing of the respective CSF samples from these patients did not detect the presence of these respiratory viruses in the central nervous system of the patients.

The demographic characteristics of infection were analyzed for all pathogens detected in >ten cases of AME using data from ‘confirmed’ or ‘highly probable’ aetiology (Table 3). JEV was more commonly detected in children aged one to ten years with a median age of five years. Whereas, DENV and CHIKV-associated AME cases were more evenly distributed amongst the age groups (median ages of six and seven, respectively). Enterovirus and *S. pneumoniae* infections were more commonly detected in younger children (median age of four for both), particularly children between one to five years. *O. tsutsugamushi* cases were more commonly associated in older children, with a median age of eight years. There was no significant difference in the gender distribution of JEV, DENV, enterovirus, *S. pneumoniae* or *O. tsutsugamushi* cases when compared to the whole study population. However, there was a highly significant gender difference in AME cases associated with CHIKV, with 16 male patients and only 1 female patient enroled.

**Figure 3:**
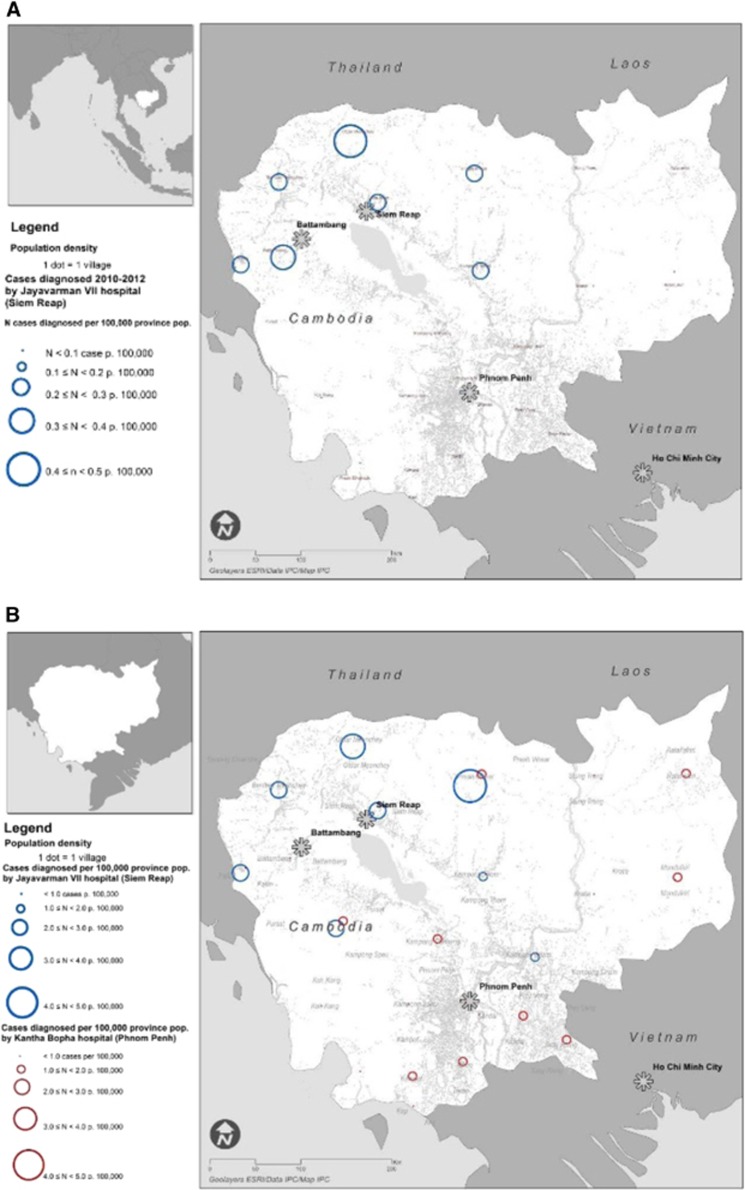
Population-adjusted attack rates (cases per 100 000 pop, using 2012 population) of Japanese encephalitis virus for the 2010–2012 study period (**A**) and 2013 study period (**B**) mapped using ArcGIS 10 (Esri, Redlands, CA, USA), by year and patient’s province of residence, to assess the time and geographical distribution of ‘confirmed’ and ‘highly probable’ cases. Population density across Cambodia was also mapped, using villages as a proxy for population (each village being represented as one dot). Note: The number of hospitals participating in the surveillance system differed between the two periods. The maps therefore use different scales to illustrate the relative incidence and burden of JEV by province during those periods rather than absolute incidence values.

**Figure 4:**
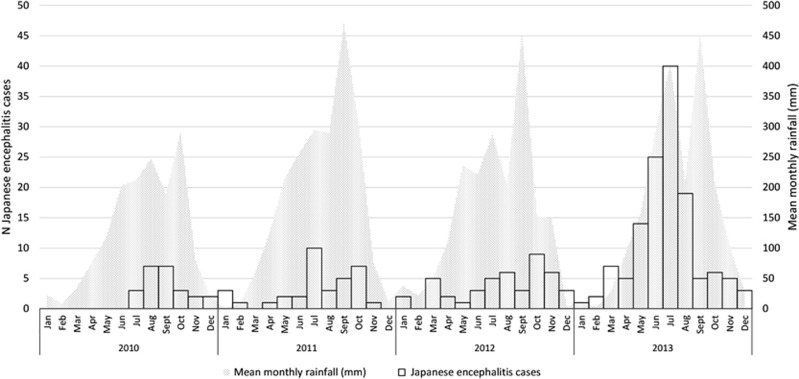
Representation of Japanese encephalitis virus cases by month with mean rainfall data estimated by satellite teledetection (Tropical Rainfall Measuring Mission, NASA, USA, http://trmm.gsfc.nasa.gov/) and adjusted for surface of province of residence.

The characteristics of JEV infection in Cambodia were analyzed to highlight the time and geographical distribution of JEV in the country and the presence of ‘hotspots’ for infection (Figures 3 and 4). Other aetiologies were not analyzed in this manner due to the much lower number of cases. JEV cases were overwhelmingly identified in the West/Northwest of Cambodia (2010–2012 recruitment in Jayavarman VII hospital, Siem Reap). When recruitment took place in both hospitals (Kantha Bopha in Phnom Penh additionally to Jayavarman VII in Siem Reap), a more comprehensive understanding of JEV epidemiology in Cambodia was obtained: cases occurred throughout the country, albeit at a lower attack rate in the South/Southwest. The population-adjusted attack rate in 2013 remained comparable to 2010–2012 in the West/Northwest of the country but Banteay Meanchey and the sparsely populated province of Preah Vihear seemed to suffer a disproportionate burden.

## DISCUSSION

The pathogens targeted for testing in this study were selected based on pre-existing data from the region on the most likely aetiologies of AME. The detection of confirmed or highly probably aetiologies in 35.0% (44.2% including ‘suspected’ cases) of cases was comparable to previous studies in Thailand (*n*=149) and Vietnam (*n*=1241) during which confirmed or probable aetiologies were identified in 36% of cases and 52% of AME patients, respectively.^16, 17^ Moreover, the NGS analysis did not identify other important pathogens that should have been included in the targeted testing. As such, we are confident that the main aetiologies of AME in Cambodia, identifiable in CSF and peripheral samples, are described.

JEV was only detected by molecular testing or culture analysis in the CSF of three patients. The difficulties of direct detection of JEV in encephalitis patients is well documented and is likely related to the low viral load in CSF and plasma during infection. Using serological and molecular testing, JEV was the most common cause of AME in this study, with 20.3% of cases ‘confirmed’ or ‘highly probable’ for JEV infection; and a further 4.1% classified as ‘suspected’. These findings support previous studies conducted in Cambodia,^11, 18, 19^ and indeed the region,^8, 17, 20, 21, 22^ reporting JEV as the main cause of infectious paediatric encephalitis. These findings should provide impetus for initiatives to further roll out JEV vaccination in the region. Currently JEV vaccination is not widespread in Cambodia. However, a nationwide JEV vaccination programme has recently been announced (1 March 2016).^23^

Mapping of JEV cases showed a backdrop of <1 diagnosed case per 100 000, with a higher burden in the North/Northwest than in the Southeast of Cambodia, which is home to 2/3 of the Cambodian population.^24^ This may be due to the Northwest being more agrarian and sparsely populated except in some urbanized Districts. It may also be due to a higher pig-raising activity in that area.^25^ Conversely, fewer cases per 100 000 pop (2012) may have been detected in the Southeast hospital network due to more diversified healthcare options in that part of Cambodia.^24^

In Southern Asia, *O. tsutsugamushi* — the agent of scrub typhus — is a common, but underappreciated cause of AME. Neurological complications have commonly been described in scrub typhus cases in India and Thailand;^26, 27, 28, 29^ and *O. tsutsugamushi* was associated with 6.1% (6/98) and 2.8% (31/1112) of AME cases in Thailand and Vietnam, respectively.^16, 30^ In the present study, *O. tsutsugamushi* was the second most common aetiology identified, with bacteremia detected by qPCR in 4.7% (*n*=55) of AME patients. In four (overall 0.3%) of these patients *O. tsutsugamushi* DNA was also detected in the CSF of the patients. These results closely reflect a study conducted in Laos that reported *O. tsutsugamushi* as the aetiology of CNS infections in 2.9% (*n*=1051) of patients by PCR or culture (detected in CSF and blood in 2.0% and 3.1% of patients, respectively).^30^ Cambodian cases were more commonly detected in older children, which is consistent with the epidemiology of scrub typhus as *O. tsutsugamushi* is transmitted through the bite of trombiculid mites (chiggers), usually found in vegetated areas. Adults and older children are therefore more likely to be infected by this organism, as previously reported in Cambodia and Vietnam.^31^ It is very important that clinicians in Cambodia and the region consider *O. tsutsugamushi* in cases of AME as the infection is readily treatable through timely and adequate antibiotic therapy.^32^

Enteroviruses and EV-A71 were detected by RT-qPCR in the CSF of 1.9% (*n*=22) and 0.8% (*n*=9) of AME cases, respectively. Suspect cases (*n*=10) of EV-A71 encephalitis were also identified through the concurrent detection of the virus in both oropharyngeal and rectal swabs from the same patient, indicating systemic infection. Prior to the enrolment of patients from Kantha Bopha Hospital in Phnom Penh, a large outbreak of encephalitis associated with EV-A71 was reported from this hospital.^33^ During this outbreak (April–July 2012) at least 56 young children died from severe encephalitis and pulmonary oedema associated with EV-A71 infection. Although fairly low numbers of EV-A71 were detected throughout the current study, the outbreak in 2012^33^ and a subsequent outbreak in 2014,^34^ highlight the importance of this pathogen in AME cases in Cambodia and the region.

Although reports of dengue infections associated with neurological symptoms have been recorded in the literature for many years,^10^ until recently it has not been generally accepted that DENV was an important cause of encephalitis. Previous studies in the Southeast Asia region have identified DENV as the aetiology of AME in 5.1% (5/99), 4.6% (9/194) and 1.3% (2/149) of cases in Cambodia,^11^ Vietnam^21^ and Thailand,^16^ respectively. In the present study, DENV was ‘confirmed’ or ‘highly probable’ in 2.4% (*n*=28) of cases (4.6% if ‘suspected’ cases are also included). Using molecular testing DENV1 (*n*=16) and DENV2 (*n*=5) were the main serotypes detected, which corresponded to a period where DENV1 and DENV2 were the predominant dengue serotypes circulating in the country.^35, 36^

The detection of CHIKV was characterized as ‘confirmed’ or ‘highly probable’ in 11 (0.9%) and six (0.5%) AME cases, respectively; a further six ‘suspected’ cases were detected through blood serology. This virus was first linked with severe neurological illnesses during the explosive outbreaks in the Indian Ocean and India during 2005–2006.^37, 38, 39^ However, CHIKV has rarely been directly detected in the CSF of AME patients. As such, the detection of CHIKV in the CSF of seven patients by RT–qPCR and/or culture in this study is noteworthy. All CHIKV AME cases were detected between June 2011 and October 2012, corresponding to a period when an outbreak of chikungunya was ongoing in Cambodia.^14, 40^ Interestingly, this outbreak was only identified following the detection of ‘sentinel’ encephalitis cases reported in the present study, leading to confirmation of the outbreak through community-based investigations.^14, 41^ The majority of CHIKV-associated AME cases were detected in males with a significant difference in the gender ratio (male:female ratio of 16). Severe CHIKV infection was more commonly observed in males in the large outbreak in India,^39^ whereas severe cases were more commonly reported in females in the Reunion Island outbreak.^37^ We do not know of any reasons why there would be such a marked difference in the gender ratio of Cambodian CHIKV AME cases as the demographic data and results from other aetiologies do not suggest a gender bias in the recruitment of cases.

The herpesviruses HSV, EBV and CMV were detected by PCR in the CSF of 0.9% (*n*=11), 0.9% (*n*=10) and 0.4% (*n*=5) of AME cases, respectively. These pathogens are commonly reported as the main aetiology of infectious AME in developed countries.^42, 43, 44, 45^ In developing countries worldwide, however, other aetiological agents such as arboviruses are more commonly detected.

The vaccine-preventable bacterial species commonly associated with meningitis, *S. pneumoniae*, *H. influenzae* type B and *N. meningitidis*, were detected in 1.6% (*n*=19), 0.7% (*n*=8) and 0.2% (*n*=2) of AME cases, respectively. These results are consistent with other studies in Vietnam, Thailand and Bangladesh with *S. pneumoniae* detected in 5.7%, 3.0% and 5.7% *H. influenzae* type B detected in 0%, 1.0% and 0.7% and *N. meningitidis* detected in 0.6%, 3.0% and 1.4% of AME cases, respectively.^16, 17, 46^ CSF bacterial culture at the hospital sites did not result in the isolation of any of these pathogens throughout the study. The high use of over-the-counter antibiotic self-medication in our setting is likely to influence isolation rates in blood and CSF cultures.^47^ *H. influenzae* type B vaccination was introduced into the Cambodian immunization schedule in 2008 and has likely resulted in a considerable reduction in cases. In contrast, the pneumococcal conjugate vaccine was introduced into the country in 2015 and the meningococcal vaccine has not yet been introduced; thus vaccination for these bacterial pathogens was not widespread during the study period, 2010–2013.

Our study has important limitations, including the lack of outcome and severity of illness data. Serological testing was focused only on arboviruses and may have led to an underestimation of other viral and bacterial causes of AME. Although tuberculosis is believed to be an important cause of AME in Cambodia the current lack of reliable and sensitive diagnostic methods for extra-pulmonary infections of *M. tuberculosis* may result in a gross underestimation of the AME burden attributable to this organism.^48^ It has been estimated that approximately one-third of all encephalitis cases are caused by immune-mediated pathogenesis.^49^ These factors were not addressed in this study and may be associated with a significant proportion of the 55.8% of cases where an aetiology was not determined.

In conclusion, our study identified JEV, *O. tsutsugamushi*, DENV, CHIKV, enteroviruses and *S. pneumoniae* as the main causes of AME in Cambodia. Therefore highlighting that the major infectious causes of AME in the country were vaccine-preventable (e.g. JEV, DENV, *S. pneumoniae*) or treatable with timely and adequate use of antimicrobials (for example, *S. pneumoniae*, *O. tsutsugamushi*). This first study to identify the major aetiologies of encephalitis in Cambodia has led to the establishment of a comprehensive project, called the Southeast Asia Encephalitis Project (SEAe, https://research.pasteur.fr/en/program_project/the-southeast-asia-encephalitis-project/), in which exhaustive laboratory identification of encephalitis pathogens, coupled with development of laboratory and clinical capacity in local hospitals is being undertaken in Cambodia, Laos, Vietnam and Myanmar. Through these initiatives we hope to increase awareness of encephalitis aetiologies among clinicians and improve laboratory capacity in hospitals to reduce the morbidity and mortality associated with AME.

## Figures and Tables

**Table 1:** Demographic characteristics of the study participants

**Characteristics**	**Siem Reap**	**Phnom Penh**	**Total**
Number of patients	931	229	1160
Date range	July 2010–September 2013	February–December 2013	July 2010–December 2013

*Age*			
Range	1 month–15 years	2 months–15 years	1 month–15 years
Median	6 years	5 years	6 years
1–11 months	36 (3.9%)	10 (4.5%)	46 (4.0%)
1–5 years	422 (45.3%)	107 (46.7%)	529 (45.6%)
6–10 years	255 (27.4%)	67 (29.3%)	322 (27.8%)
11–15 years	217 (23.3%)	40 (17.5%)	257 (22.2%)
Unknown	1 (0.1%)	5 (2.2%)	6 (0.5%)

*Sex*			
Female	381 (40.9%)	102 (44.5%)	483 (41.6%)
Male	550 (59.1%)	126 (55.0%)	676 (58.3%)
Unknown	0	1 (0.4%)	1 (0.09%)

**Table 2:** Laboratory results to determine the aetiology of acute meningoencephalitis in Cambodian children

**Pathogen**^a^	**Confirmed**			**Highly probable** **CSF IgM or PCR detection of CMV or EBV in CSF**	**Suspected** **Blood IgM or EV71 throat and rectal PCR**	**Total (n=1,160)**
	**CSF—PCR or Culture**	**Blood—PCR or Culture**	**Total**			
Japanese encephalitis virus	3	0	3 (0.3%)	233 (20.1%)	47 (4.1%)	283 (24.4%)
Dengue virus 1	10^b^	6^b^	16 (4.4%)			
Dengue virus 2	3^c^	2^c^	5 (0.4%)	5 (0.4%)	25 (2.2%)	53 (4.6%)
Dengue virus 3	0	1	1 (0.09%)			
Dengue virus 4	0	1	1 (0.09%)			
Flavivirus	0	0	0	8 (0.7%)	20 (1.7%)	28 (2.4%)
Chikungunya virus	7^d^	4^d^	11 (0.9%)	6 (0.5%)	6 (0.5%)	23 (2.0%)
Enterovirus	22	0	22 (1.9%)	NA	NA	22 (1.9%)
Enterovirus 71	9	0	9 (0.8%)	NA	10 (0.9%)	19 (1.6%)
Herpes simplex virus	11	NA	11 (0.9%)	NA	NA	11 (0.9%)
Epstein-Barr virus	NA	NA	NA	10 (0.9%)	NA	10 (0.9%)
Cytomegalovirus	NA	NA	NA	5 (0.4%)	NA	5 (0.4%)
*Streptococcus pneumoniae*	19	0	19 (1.6%)	NA	NA	19 (1.6%)
*Haemophilus influenzae*	8	0	8 (0.7%)	NA	NA	8 (0.7%)
*Neisseria meningitidis*	2	0	2 (0.2%)	NA	NA	2 (0.2%)
*Orientia tsutsugamushi*	4^e^	51^e^	55 (4.7%)	NA	NA	55 (4.7%)
Total^f^	NA	NA	141 (12.2%)	265 (22.8%)	107 (9.2%)	513 (44.2%)

Abbreviations: cytomegalovirus, CMV; cerebrospinal fluid, CSF; Epstein-Barr virus, EBV; immunoglobulin, IgM; not applicable, NA.

a Only pathogens detected in the study are included in the table.

b 2 cases were positive in both CSF and blood, 8 cases were positive in CSF only, 6 cases were positive in blood only.

c 1 case was positive in both CSF and blood, 2 cases were positive in CSF only, 2 cases were positive in blood only.

d 6 cases were positive in both CSF and blood, 1 case was positive in CSF only, 4 cases were positive in blood only.

e 4 cases were positive in both CSF and blood, 0 cases were positive in CSF only, 51 cases were positive in blood only.

f Only the number of patients were included in the total amounts (i.e. a coinfection with two pathogens was not counted twice).

**Table 3:** Age and gender characteristics for patients in which the main pathogens were detected

**Pathogen detected**^a^	**Gender ratio (M:F)**	**Median age (years)**	**Age groups (% of total number in age group)**			
			**1–11 months**	**1–5 years**	**6–10 years**	**11–15 years**
JEV	1.6 (144:92)	5	1 (2.2%)	119 (22.5%)	85 (26.4%)	30 (11.7%)
DENV1-4	1.5 (17:11)	6	2 (4.3%)	12 (2.3%)	9 (2.8%)	5 (1.9%)
CHIKV	16 (16:1)	7	0	8 (1.5%)	4 (1.2%)	5 (1.9%)
Enterovirus^b^	1.8 (20:11)	4	0	19 (3.6%)	9 (2.8%)	3 (1.2%)
*S. pneumoniae*	1.4 (11:8)	4	0	12 (2.3%)	4 (1.2%)	3 (1.2%)
*O. tsutsugamushi*	1.4 (32:23)	8	1 (2.2%)	18 (3.4%)	11 (3.4%)	25 (9.7%)
Baseline	1.4 (676:483)	6	*n*=46	*n*=529	*n*=322	*n*=257

Abbreviations: chikungunya virus, CHIKV; dengue virus, DENV; Japanese encephalitis virus, JEV.

a Confirmed and highly probable aetiologies.

b Including EV-A71.

## References

[bib1] Wills B, Farrar J. Central nervous system infections in the tropics: diagnosis, treatment and prevention. Curr Opin Infect Dis 2000; 13: 259–264. 11964796 10.1097/00001432-200006000-00010

[bib2] Furyk JS, Swann O, Molyneux E. Systematic review: neonatal meningitis in the developing world. Trop Med Int Health 2011; 16: 672–679. 21395927 10.1111/j.1365-3156.2011.02750.x

[bib3] Briand C, Levy C, Baumie F et al. Outcomes of bacterial meningitis in children. Med Mal Infect 2016; 46: 177–187. 27020729 10.1016/j.medmal.2016.02.009

[bib4] Global Burden of Disease Study 2013 Collaborators. Global, regional, and national incidence, prevalence, and years lived with disability for 301 acute and chronic diseases and injuries in 188 countries, 1990–2013: a systematic analysis for the Global Burden of Disease Study 2013. Lancet 2015; 386: 743–800. 26063472 10.1016/S0140-6736(15)60692-4 PMC4561509

[bib5] Global Burden of Disease Study 2013 Collaborators. Global, regional, and national age-sex specific all-cause and cause-specific mortality for 240 causes of death, 1990–2013: a systematic analysis for the Global Burden of Disease Study 2013. Lancet 2015; 385: 117–171. 25530442 10.1016/S0140-6736(14)61682-2 PMC4340604

[bib6] Whitley RJ, Gnann JW. Viral encephalitis: familiar infections and emerging pathogens. Lancet 2002; 359: 507–513. 11853816 10.1016/S0140-6736(02)07681-X

[bib7] Campbell GL, Hills SL, Fischer M et al. Estimated global incidence of Japanese encephalitis: a systematic review. Bull World Health Organ 2011; 89: 766–774. 22084515 10.2471/BLT.10.085233 PMC3209971

[bib8] Tarantola A, Goutard F, Newton P et al. Estimating the burden of Japanese encephalitis virus and other encephalitides in countries of the mekong region. PLoS Negl Trop Dis 2014; 8: e2533. 24498443 10.1371/journal.pntd.0002533 PMC3907313

[bib9] The World BankCountry at a Glance: Cambodia. The World Bank Group: Washington, DC. 2016. Available at http://data.worldbank.org/country/cambodia (accessed on 1 August 2016).

[bib10] Solomon T, Dung NM, Vaughn DW et al. Neurological manifestations of dengue infection. Lancet 2000; 355: 1053–1059. 10744091 10.1016/S0140-6736(00)02036-5

[bib11] Srey VH, Sadones H, Ong S et al. Etiology of encephalitis syndrome among hospitalized children and adults in Takeo, Cambodia, 1999–2000. Am J Trop Med Hyg 2002; 66: 200–207. 12135294 10.4269/ajtmh.2002.66.200

[bib12] Rossi CA, Ksiazek TG. Enzyme-linked immunosorbent assay (ELISA). In: Lee HW, Calisher C, Schmaljohn C (eds). Manual of Hemorrhagic Fever with Renal Syndrome and Hantavirus Pulmonary Syndrome. Seoul: WHO Collaborating Center for Virus Reference and Research, 1998: 87–91.

[bib13] Vong S, Khieu V, Glass O et al. Dengue incidence in urban and rural Cambodia: results from population-based active fever surveillance, 2006–2008. PLoS Negl Trop Dis 2010; 4: e903. 21152061 10.1371/journal.pntd.0000903 PMC2994922

[bib14] Ly S, Sorn S, Tarantola A et al. Chikungunya outbreak—Cambodia, February–March 2012. MMWR Morb Mortal Wkly Rep 2012; 61: 737–740. 22992571

[bib15] Gagnieur L, Cheval J, Gratigny M et al. Unbiased analysis by high throughput sequencing of the viral diversity in fetal bovine serum and trypsin used in cell culture. Biologicals 2014; 42: 145–152. 24661556 10.1016/j.biologicals.2014.02.002

[bib16] Olsen SJ, Campbell AP, Supawat K et al. Infectious causes of encephalitis and meningoencephalitis in Thailand, 2003–2005. Emerg Infect Dis 2015; 21: 280–289. 25627940 10.3201/eid2102.140291 PMC4313633

[bib17] Ho Dang Trung N, Le Thi Phuong T, Wolbers M et al. Aetiologies of central nervous system infection in Viet Nam: a prospective provincial hospital-based descriptive surveillance study. PLoS One 2012; 7: e37825. 22662232 10.1371/journal.pone.0037825 PMC3360608

[bib18] Chhour YM, Ruble G, Hong R et al. Hospital-based diagnosis of hemorrhagic fever, encephalitis, and hepatitis in Cambodian children. Emerg Infect Dis 2002; 8: 485–489. 11996683 10.3201/eid0805.010236 PMC2732496

[bib19] Touch S, Hills S, Sokhal B et al. Epidemiology and burden of disease from Japanese encephalitis in Cambodia: results from two years of sentinel surveillance. Trop Med Int Health 2009; 14: 1365–1373. 19747185 10.1111/j.1365-3156.2009.02380.x

[bib20] Hossain MJ, Gurley ES, Montgomery S et al. Hospital-based surveillance for Japanese encephalitis at four sites in Bangladesh, 2003–2005. Am J Trop Med Hyg 2010; 82: 344–349. 20134015 10.4269/ajtmh.2010.09-0125 PMC2813179

[bib21] Le VT, Phan TQ, Do QH et al. Viral etiology of encephalitis in children in southern Vietnam: results of a one-year prospective descriptive study. PLoS Negl Trop Dis 2010; 4: e854. 21049060 10.1371/journal.pntd.0000854 PMC2964288

[bib22] Feng Y, Fu S, Zhang H et al. High incidence of Japanese encephalitis, southern China. Emerg Infect Dis 2013; 19: 672–673. 23750863 10.3201/eid1904.120137 PMC5836486

[bib23] United NationsChildren's Emergency Fund (UNICEF)Nation-Wide Japanese Encephalitis Vaccination Campaign Launched in Cambodia and to be Introduced into Routine Immunization Schedule. UNICEF: Phnom Penh. 2016. Available at http://www.unicef.org/cambodia/12681_25295.html (accessed on 10 May 2016).

[bib24] Annear PL, Grundy J, Ir P, Jacobs B, Men C, Nachtnebel M, Oum S, Robins A, Ros CE. The Kingdom of Cambodia Health System Review. Health Systems in Transition. World Health Organization: Geneva. 2015 Available at http://www.wpro.who.int/asia_pacific_observatory/hits/series/cambodia_health_systems_review.pdf (accessed on 2 August 2016).

[bib25] Food and Agriculture Organization of the United Nations (FAO), Regional Office For Asia and the PacificSwine Industry Profile of Selected South East Asian Countries. Cambodia, Lao PDR, Myanmar, Philippines, Thailand, Vietnam. Bangkok: FAO. 2011 Available at http://cdn.aphca.org/dmdocuments/PAP_11_SE%20Asia%20Pig%20Industry%20Profiles_TCP%20RAS%203215.pdf (accessed on 1 August 2016).

[bib26] Silpapojakul K, Ukkachoke C, Krisanapan S, Silpapojakul K. Rickettsial meningitis and encephalitis. Arch Intern Med 1991; 151: 1753–1757. 1888241

[bib27] Mahajan SK, Rolain JM, Kanga A, Raoult D. Scrub typhus involving central nervous system, India, 2004–2006. Emerg Infect Dis 2010; 16: 1641–1643. 20875303 10.3201/eid1610.100456 PMC3294396

[bib28] Misra UK, Kalita J, Mani VE. Neurological manifestations of scrub typhus. J Neurol Neurosurg Psychiatry 2015; 86: 761–766. 25209416 10.1136/jnnp-2014-308722

[bib29] Sharma SR, Masaraf H, Lynrah KG, Lyngdoh M. Tsutsugamushi disease (scrub typhus) meningoencephalitis in north eastern India: A prospective study. Ann Med Health Sci Res 2015; 5: 163–167. 26097756 10.4103/2141-9248.157486 PMC4455004

[bib30] Dittrich S, Rattanavong S, Lee SJ et al. Orientia, rickettsia, and leptospira pathogens as causes of CNS infections in Laos: a prospective study. Lancet Glob Health 2015; 3: e104–e112. 25617190 10.1016/S2214-109X(14)70289-X PMC4547322

[bib31] Duong V, Mai TT, Blasdell K et al. Molecular epidemiology of *Orientia tsutsugamushi* in Cambodia and Central Vietnam reveals a broad region-wide genetic diversity. Infect Genet Evol 2013; 15: 35–42. 21241829 10.1016/j.meegid.2011.01.004

[bib32] Peter JV, Sudarsan TI, Prakash JA, Varghese GM. Severe scrub typhus infection: Clinical features, diagnostic challenges and management. World J Crit Care Med 2015; 4: 244–250. 26261776 10.5492/wjccm.v4.i3.244 PMC4524821

[bib33] Doung V, Mey C, Eloit M et al. Molecular epidemiology of human enterovirus 71 at the origin of an epidemic of fatal hand, foot and mouth disease cases in Cambodia. Emerg Microbes Infect 2016; 5: e104. 27651091 10.1038/emi.2016.101 PMC5113052

[bib34] Horwood PF, Andronico A, Tarantola A et al. Seroepidemiology of Human Enterovirus 71 Infection among Children, Cambodia. Emerg Infect Dis 2016; 22: 92–95. 26690000 10.3201/eid2201.151323 PMC4696711

[bib35] Lover AA, Buchy P, Rachline A et al. Spatial epidemiology and climatic predictors of paediatric dengue infections captured via sentinel site surveillance, Phnom Penh Cambodia 2011–2012. BMC Public Health 2014; 14: 658. 24972712 10.1186/1471-2458-14-658 PMC4085229

[bib36] Duong V, Lambrechts L, Paul RE et al. Asymptomatic humans transmit dengue virus to mosquitoes. Proc Natl Acad Sci USA 2015; 112: 14688–14693. 26553981 10.1073/pnas.1508114112 PMC4664300

[bib37] Economopoulou A, Domínguez M, Helynck B et al. Atypical chikungunya virus infections: clinical manifestations, mortality and risk factors for severe disease during the 2005–2006 outbreak on Réunion. Epidemiol Infect 2009; 137: 534–541. 18694529 10.1017/S0950268808001167

[bib38] Gérardin P, Couderc T, Bintner M et al. Chikungunya virus-associated encephalitis: A cohort study on La Réunion Island, 2005-2009. Neurology 2016; 86: 94–102. 26609145 10.1212/WNL.0000000000002234

[bib39] Tandale BV, Sathe PS, Arankalle VA et al. Systemic involvements and fatalities during Chikungunya epidemic in India, 2006. J Clin Virol 2009; 46: 145–149. 19640780 10.1016/j.jcv.2009.06.027

[bib40] Duong V, Andries AC, Ngan C et al. Reemergence of Chikungunya virus in Cambodia. Emerg Infect Dis 2012; 18: 2066–2069. 23171736 10.3201/eid1812.120471 PMC3557864

[bib41] Galatas B, Ly S, Duong V et al. Long-Lasting Immune Protection and Other Epidemiological Findings after Chikungunya Emergence in a Cambodian Rural Community, April 2012. PLoS Negl Trop Dis 2016; 10: e0004281. 26752630 10.1371/journal.pntd.0004281 PMC4713465

[bib42] Glaser CA, Gilliam S, Schnurr D et al. In search of encephalitis etiologies: diagnostic challenges in the California Encephalitis Project, 1998–2000. Clin Infect Dis 2003; 36: 731–742. 12627357 10.1086/367841

[bib43] Mailles A, Stahl JP Steering Committee and Investigators Group. Infectious encephalitis in France in 2007: a national prospective study. Clin Infect Dis 2009; 49: 1838–1847. 19929384 10.1086/648419

[bib44] Granerod J, Ambrose HE, Davies NW et al. Causes of encephalitis and differences in their clinical presentations in England: a multicentre, population-based prospective study. Lancet Infect Dis 2010; 10: 835–844. 20952256 10.1016/S1473-3099(10)70222-X

[bib45] Granerod J, Cousens S, Davies NW, Crowcroft NS, Thomas SL. New estimates of incidence of encephalitis in England. Emerg Infect Dis 2013; 19: 1455–1462. 10.3201/eid1909.130064 PMC3810913 23969035

[bib46] Rasul CH, Muhammad F, Hossain MJ, Ahmed KU, Rahman M. Acute meningoencephalitis in hospitalised children in southern Bangladesh. Malays J Med Sci 2012; 19: 67–73. 22973139 PMC3431749

[bib47] Om C, McLaws M-L, Vlieghe E, Daily F, McLaughlin JC. Cambodia: the first national study of antibiotic prescribing and resistance using mixed methods approach. Antimicrob Resist Infect Control 2015; 4 (S1): 183.

[bib48] Schoeman JF, Donald PR. Tuberculous meningitis. Handb Clin Neurol 2013; 112: 1135–1138. 23622321 10.1016/B978-0-444-52910-7.00033-7

[bib49] Granerod J, Cunningham R, Zuckerman M et al. Causality in acute encephalitis: defining aetiologies. Epidemiol Infect 2010; 138: 783–800. 20388231 10.1017/S0950268810000725

